# Experimental and theoretical investigation of the influence of post-curing on mixed mode fracture properties of 3d-printed polymer samples

**DOI:** 10.1038/s41598-024-64136-y

**Published:** 2024-06-08

**Authors:** Bahador Bahrami, Hossein Talebi, M. M. Momeni, M. R. Ayatollahi

**Affiliations:** Fatigue and Fracture Research Laboratory, Center of Excellence in Experimental Solid Mechanics and Dynamics, School of Mechanical Engineering, Iran University of Science and Technology, Narmak, Tehran, 16846 Iran

**Keywords:** Acrylonitrile butadiene styrene (ABS), Heat treatment, Maximum tangential stress (MTS), Equivalent material concept (EMC), Mechanical engineering, Mechanical properties

## Abstract

This study explores the mechanical properties and fracture characteristics of additively manufactured acrylonitrile butadiene styrene specimens, focusing on the impact of raster angle and post-process heat treatment. To this end, a large number of tensile and semi-circular bending samples with three distinct raster angles of 0/90°, 22/ − 68°, and 45/ − 45° were prepared and exposed to four types of heat treatments with different temperature and pressure conditions. Simultaneously, theoretical models of maximum tangential stress (MTS) and generalized MTS (GMTS) were developed to estimate the onset of specimen fracture under mixed-mode in-plane loading conditions. Recognizing the non-linear behavior within the stress–strain curve of tensile test samples, particularly in the annealed samples, an effort was undertaken to transform the original ductile material into a virtual brittle material through the application of the equivalent material concept (EMC). This approach serves the dual purpose of bypassing intricate and tedious elastoplastic analysis, while concurrently enhancing the precision of the GMTS criterion. The experimental findings have revealed that while the annealing process has a minimal effect on the yield strength, it considerably enhances energy absorption capacity, increases fracture toughness, and reduces the anisotropy. Additionally, the combined EMC-GMTS criterion has demonstrated its capability to predict the failure of the additively manufactured parts with an acceptable level of accuracy.

## Introduction

Modern manufacturing is being revolutionized by a pioneering technology that has changed the way we conceive, design, and produce objects of all scales and complexities. The concept of additive manufacturing (AM), often referred to as 3D printing, represents a revolutionary departure from subtractive manufacturing, which involves removing raw materials to form a final product. Through AM, layers of material are built up to create intricate and highly customized designs that were once thought to be unattainable. In diverse industries, such as aerospace^1^, automotive^2^, and healthcare^3^, this transformative approach has found widespread applications. The process begins with a digital design, which is then translated into physical reality using a variety of materials, ranging from plastics and metals to ceramics. The versatility of materials, combined with the ability to produce sophisticated geometries without excessive waste, makes 3D printing both eco-friendly and cost-effective.

In terms of tools and techniques, different AM methods have been developed up to now, which are classified into seven categories, including sheet lamination, powder bed fusion, material jetting, direct energy deposition, vat photopolymerization, material extrusion, and binder jetting^4^. In fused deposition modeling (FDM), a prominent subset of material extrusion, thermoplastic materials are delicately guided through a heated nozzle, where they undergo controlled melting before being precisely deposited layer-by-layer onto a build surface.

Exploring the viability of additively manufactured components for real-world applications necessitates a comprehensive knowledge of their mechanical characteristics. This is because the properties of 3D-printed objects can vary depending on the type of material used, the build parameters, and the post-processing techniques employed. Consequently, researchers have explored the intricate effects of manufacturing parameters. An array of factors contributes to these effects, including but not limited to build orientation^5–7^, layer thickness^8,9^, feed rate^10,11^, plate temperature^12,13^, raster angle^14–16^, air gap^17,18^, extruder temperature^19,20^, and color^21^.

Various methodologies have been presented to improve the mechanical properties of 3D-printed parts. Material enhancement, structural optimization, and post-print processing are all involved in these techniques with the aim of improving the structural integrity, durability, and functional versatility of additively manufactured parts. Through the synergy of innovation and engineering, these methods offer a path to overcome the inherent limitations of 3D printing and unlock a new level of excellence in mechanical design. As one of the most significant post-processing techniques, heat treatment is a valuable tool for enhancing the mechanical characteristics of fabricated components. Through the interactions between temperature, time, and controlled thermal cycles during heat treatment, microstructural transformation changes can be induced, which affects characteristics such as strength, toughness, and even dimensional stability. In a recent study, Szust and Adamski^22^ demonstrated that subjecting FDM polylactic acid (PLA) samples to heat treatment at 60 °C for an hour leads to a remarkable 24% improvement in strength. Furthermore, even after undergoing the annealing process, the anisotropy of the 3D-printed samples remained notably significant. Bhandari and collaborators^23^ demonstrated that subjecting polyethylene terephthalate-glycol carbon fiber (PETG-CF) and PLA-CF composite materials to heat treatment at an optimal temperature leads to a substantial improvement in their interlayer tensile strength. Using acrylonitrile butadiene styrene (ABS) and PLA additive manufacturing parts, Lluch-Cerezo et al.^24^ investigated how heat treatment temperature affects ceramic powder mold performance. The findings revealed that the effectiveness of the mold diminishes when dealing with parts fabricated from PLA. In comparison to amorphous polymers like ABS, PLA is a semi-crystalline thermoplastic, which shrinks less during thermal post-processing. The test of flexural strength showed that the mold exerts negligible influence on the mechanical properties of the parts.

This research aims to comprehensively investigate how raster angle and post-processing heat treatment impact the mechanical properties and fracture behavior of ABS samples. This was done through experimental tests and theoretical model development. Initially, 45 tensile test samples and 180 semi-circular bending (SCB) samples (A renowned sample to extract fracture properties of materials) were fabricated utilizing the FDM technique with three distinct raster angles. Subsequently, both heat-treated and as-printed SCB samples were subjected to combined in-plane loading, during which their fracture load values and crack propagation patterns were recorded. In the next stage, the fracture loads obtained from experimental tests were utilized in finite element (FE) models to calculate critical stress intensity factors (SIFs). In parallel, a theoretical investigation was conducted to predict the fracture behavior of these samples. Fracture limit curves were developed for additively manufactured ABS samples using the linear elastic maximum tangential stress (MTS) and generalized MTS (GMTS) criteria in combination with the equivalent material concept (EMC). This concept generates an equivalent ultimate tensile strength which is then combined with the GMTS criterion to more accurately predict fracture behavior. Ultimately, to assess the consistency and correspondence between the empirical findings and theoretical predictions, the empirical results were compared to the theoretical predictions.

## Experimental procedure

This section provides an overview of the process employed to fabricate the test specimens, as well as a detailed description of the subsequent experimental tests conducted. Experiments were carried out to analyze stress–strain curves from diverse tensile test samples and fracture patterns in SCB samples under different in-plane loading conditions. This aimed to identify the influence of raster angle and heat treatment on fracture behavior and stress–strain characteristics.

### Specimen fabrication

In the preliminary phase, tensile and fracture test samples were designed through computer-aided design software and subsequently fed to an FDM 3D printer device. The chosen material for these specimens was ABS, a material renowned for its mechanical robustness^25,26^. The filament used was named ABS + 3D-printer filament and was produced by MagicFilament company.

**Figure 1:**
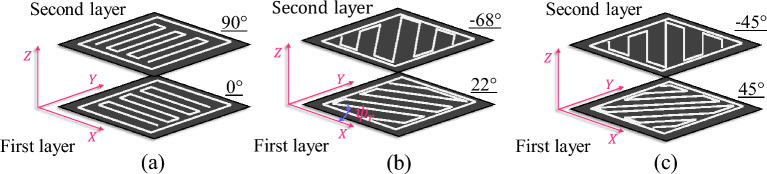
Schematic of the build orientation at three raster angles of (**a**) 0/90°, (**b**) 22/ − 68°, and (**c**) 45/ − 45°.

Given the multifaceted scope of this study, which explores not only the effects of heat treatment but also the influence of raster angle, a comprehensive approach was undertaken. To achieve this, samples were printed with three distinct raster angles of $\psi_{{\text{r}}} = 0/90^\circ$, $22/ - 68^\circ$, and $45/ - 45^\circ$. Figure 1 gives a representation of these raster builds. The FDM construction parameters for fabricating the tensile and fracture test specimens are summarized in Table 1. As it is clear from this table, since the infill density of the specimens is taken as 100%, the value for the programmed air gap in the G-code should be equal to zero. Note that this value differs from the actual air gap that might occur in between rasters. Nozzle and bed temperatures were set to the recommended values by the filament manufacturer. A moderate printing speed of 60 mm/s was taken constant for the production of all samples. Raster width and layer height are the width and thickness of the raster profile cross-section. Upon completion of the printing process, the specimens were allowed to rest on the printing bed for a cooling period of 30 min. To mitigate any potential warping or distortion that could compromise the structural integrity of samples, this cooling step was vital. Studies show that bed temperature plays an important role in warpage prevention of FDM materials^26,27^.

**Table 1:** Constant parameters of the FDM 3D printing process.

Construction parameters	Nozzle temperature (°C)	Bed temperature (°C)	Printing speed (mm/s)	Raster width (mm)	Layer height (mm)	Programmed air gap (mm)	Infill density (%)
Value	240	110	60	0.5	0.25	0	100

### Heat treatment

**Figure 2:**
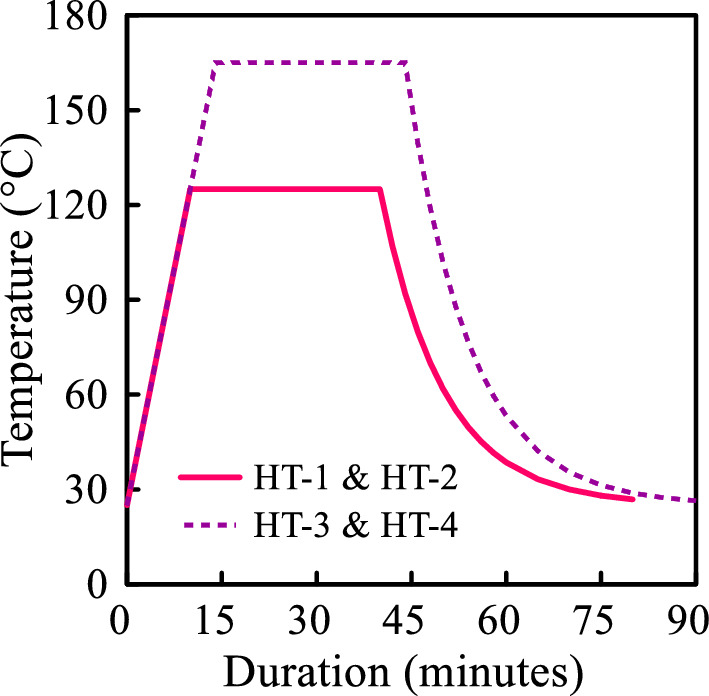
Temperature changes during the annealing process.

The use of heat treatment is one of the most prominent post-processing techniques in AM. This research focuses on the profound influence of heat treatment on mechanical properties as well as the fracture behavior of ABS additively manufactured parts. Accordingly, the process of thermal treatment was executed with the application of distinct temperature and pressure settings. Table 2 presents the temperature, pressure, and duration pertaining to each individual heat treatment process, while Fig. 2 graphically depicts the temperature transformations that occurred throughout the annealing process. The temperature of the furnace was kept constant using the default temperature control system, which uses a thermal sensor in the heat treatment chamber to control the temperature. The furnace is also equipped with a fan, unifying the temperature inside. Unlike temperature, pressure is not continuously monitored, however, acknowledging the uniform distribution of the weight of the aluminum sheets on the specimens, it is concluded that pressure was kept uniform and almost constant during the heat treatment process.

**Table 2:** The parameters of four different heat treatment processes.

Annealing process code	HT-1	HT-2	HT-3	HT-4
Maximum temperature (°C)	125	125	165	165
Pressure (kPa)	0.58	1.52	0.58	1.52
Maximum temperature duration (minutes)	30	30	30	30

These parameters are carefully chosen to exceed the glass transition temperature (*T*_g_ = 105 °C for ABS) while staying below the melting point of the material. The purpose of this deliberate selection is to allow the material to undergo favorable transformations without suffering structural degradation due to excessive heat. On the other hand, annealing for ABS serves as a method to achieve a more uniform surface texture for printed models while effectively eliminating imperfections like the thread-like lines that can arise during the printing process.

### Tensile test

**Figure 3:**
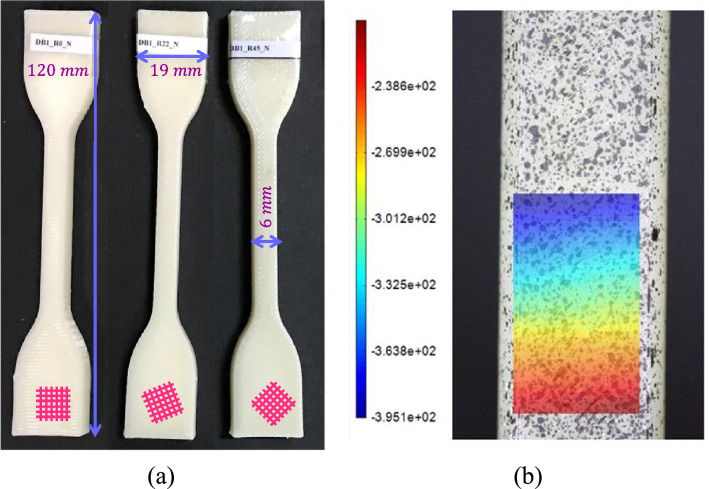
(**a**) Simple tensile test specimens made in three different raster orientations. (**b**) Vertical displacement field from DIC (in pixels).

A simple tensile test was conducted according to type IV of the ASTM-D638 standard^28^ to measure the mechanical characteristics of printed components. Thus, a total of 45 dog-bone samples were fabricated. Out of this batch, 9 specimens were selected for testing in their original state, devoid of any post-processing treatment, while the rest of the samples were exposed to controlled thermal conditions within a furnace based on the heat treatment processes listed in Table 2 prior to being subjected to mechanical testing. Figure 3a shows the as-printed tensile test samples made at three different raster configurations. While the FDM 3D-printing machine used to fabricate the specimens had high precision, we ensured the accuracy of the dimensions of the tensile and SCB specimens by measuring each specimen with a digital caliper with a precision of 0.01 mm. The measured values were then used for further calculations and FE modeling. This process ensured the reliability and accuracy of our experimental data.

The test specimens were exposed to tensile forces utilizing a universal tensile testing device, with a consistent displacement rate of $0.5\frac{{{\text{mm}}}}{{{\text{min}}}}$. Concurrently, the digital image correlation (DIC) technique was employed to provide a higher degree of accuracy in recording strain measurements. Using the DIC technique ensures that the area with the highest plastic deformation within the tensile specimen was captured and its strain measurements were recorded. The average strain in this area would then be used as the strain data for each tensile test, which was necessary for a valid stress–strain curve to be later used in the EMC. A subset size of 100 and a step size of 15 pixels were used for the DIC process. Figure 3b depicts the vertical displacement field obtained from one of the heat-treated tensile samples as an instance. The symmetry between the left and right sides of strain contours represents uniform tension across the specimen width during the test. It is pertinent to emphasize that each tensile test was repeated three times to ensure the reproducibility and consistency of the outcomes.

### Fracture test

**Figure 4:**
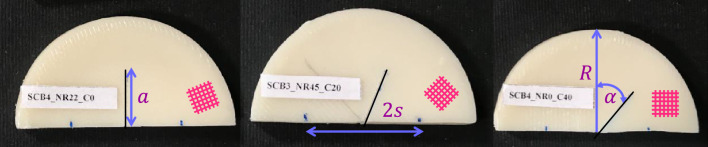
SCB samples made in three different raster angles and three distinct crack angles.

Samples were originally printed as discs with a diameter of 50 mm and an initial thickness of 8 mm. Then they were cut by a hacksaw into two halves at desired angles to obtain SCB specimens. The reason behind choosing this manufacturing method was to remove the barrel-shaped surfaces created during the heat treatment. This ensures that the SCB’s support locations are flat. As a next step, 12.5 mm cracks were created on half-disk samples. However, the main challenge in making cracked samples was ensuring the crack had a sharp tip. To overcome this, we followed a two-step procedure: first, we used a hacksaw to cut 10.5 mm, and then we meticulously crafted the final 2 mm using a fresh razor blade. By using this approach, we were able to conquer the challenge and achieve well-defined crack tips. Cracks were purposefully created on the samples at three different angles: 0°, 20°, and 40°. A deliberate variation in angles was intended to cover not only pure mode I loading, but also combined mode I/II and pure mode II loading conditions. Taking advantage of this range of angles, the study explored the diverse responses of SCB samples to different loading conditions in order to acquire a good understanding of fracture mechanisms. Cracked samples were produced at three different raster angles and exposed to the four specified heat treatment conditions detailed in Sect. “Experimental procedure”, with each test conducted four times, resulting in a total of 180 fracture test specimens comprising 36 as-printed and 144 annealed samples. Through repetition and variations in conditions, a wide range of datasets for analyzing the fracture characteristics of 3D-printed ABS specimens was established. Three SCB samples fabricated for pure mode I, combined mode I/II, and pure mode II loading conditions are shown in Fig. 4.

**Figure 5:**
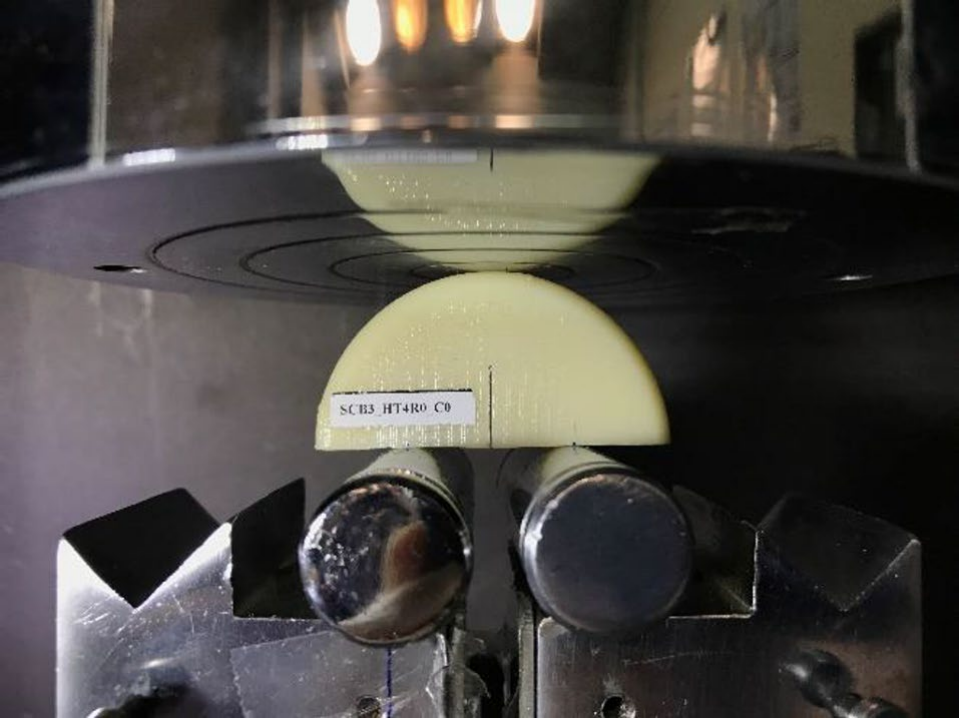
Mode I SCB specimen and its loading set-up.

According to loading and boundary conditions, the force was applied with a constant displacement rate of 0.5 mm/min to the upper portion of the specimen through a flat disc, while two free rollers supported the lower portion which is shown in Fig. 5^29,30^. The distance between the two supports (2*S*) was set at 25 mm. The dimensions of all SCB samples were measured after production. The crack angle and crack length were meticulously measured using a protractor and caliper to ensure their accuracy. These measured values were then used for each specimen in the FE analysis to obtain accurate values of SIFs and T-stress.

## Theoretical analyses

In this study, alongside experimental investigations into fracture behavior, theoretical models were developed to draw fracture limit curves for all fracture angles. These models were then assessed for accuracy against the experimental results. In the following, we will explore the methodology that was employed to derive theoretical models.

The SCB specimen is a well-known geometry employed frequently in the past to investigate mixed-mode in-plane fracture phenomena. The mathematical equations for the critical SIFs ($K_{If}$ and $K_{IIf}$) and the T-stress at the fracture point $\left( {T_{f} } \right)$ for the SCB specimen are as follows.

1 $$ K_{If} = Y_{I} \frac{{P_{cr} }}{2Rt}\sqrt {\pi a} $$

2 $$ K_{IIf} = Y_{II} \frac{{P_{cr} }}{2Rt}\sqrt {\pi a} $$

3 $$ T_{f} = T^{*} \frac{{P_{cr} }}{2Rt} $$

where $Y_{I}$ and $Y_{II}$ are geometric factors and their values were obtained through FE modeling. $T^{*}$ is the dimensionless form of the T-stress term; $P_{cr}$ stands for the fracture load; $t$ denotes the thickness of the sample and other parameters are displayed in Fig. 4. In the following subsections, a brief overview of the theories employed in this study is provided.

### MTS and GMTS criteria

In the context of linear elastic fracture mechanics, a wide range of criteria has been developed to predict the fracture of materials exhibiting brittle behavior. These criteria can be categorized into two distinct classes: energy-based and stress-based criteria. In addition to providing a comprehensive understanding of the underlying mechanics governing brittle failure, these theoretical models are critical analytical tools for assessing material vulnerability under specific loading conditions.

In the scope of this study, we employed a renowned stress-based criterion, specifically the maximum tangential stress (MTS) linear elastic criterion. The MTS criterion, in the context of combined-mode loading conditions, implies that the pre-existing crack commences propagation perpendicular to the direction of the utmost tangential stress. This progression occurs when the tangential stress, precisely at a critical distance, denoted as $r_{c}$, in advance of the crack tip, meets the material critical stress $\sigma_{c}$ threshold. To enhance the precision of the MTS approach, Smith et al.^31^ introduced the generalized form of the MTS (GMTS) theoretical model. Within this criterion, the T-stress term is integrated alongside the singular terms of the Williams infinite series expansion. Thus, the tangential stress equation is modified as follows^32^.

4 $$ \sigma_{\theta \theta } = \frac{1}{{\sqrt {2\pi r} }}\cos \frac{\theta }{2}\left[ {K_{I} \cos^{2} \frac{\theta }{2} - \frac{3}{2}K_{II} \sin \theta } \right] + T\sin^{2} \theta $$

where $$r$$ and $$\theta$$ are polar coordinate parameters in which the origin is at the crack tip;$$ K_{I}$$ and $$K_{II}$$ represent the mode I and II SIFs.

Here, we present only the equations associated with the GMTS criterion. Nevertheless, due to the fundamental similarity underpinning these two theoretical models, a straightforward substitution of zero for the T-stress term in all equations readily yields the corresponding MTS criterion equations. According to the assumptions of GMTS criterion, the following equations can be derived.

5 $$ \frac{{K_{If} }}{{K_{Ic} }} = \left[ {\cos \frac{{\theta_{0} }}{2}\left( {\cos^{2} \frac{{\theta_{0} }}{2} - \frac{3}{2}\frac{{Y_{II} }}{{Y_{I} }}\sin \theta_{0} } \right) + \sqrt {\frac{{2r_{c} }}{a}} \frac{{T^{*} }}{{Y_{I} }}\sin^{2} \theta_{0} } \right]^{ - 1} $$

6 $$ \frac{{K_{IIf} }}{{K_{Ic} }} = \left[ {\cos \frac{{\theta_{0} }}{2}\left( {\frac{{Y_{I} }}{{Y_{II} }}\cos^{2} \frac{{\theta_{0} }}{2} - \frac{3}{2}\sin \theta_{0} } \right) + \sqrt {\frac{{2r_{c} }}{a}} \frac{{T^{*} }}{{Y_{II} }}\sin^{2} \theta_{0} } \right]^{ - 1} $$

Detailed information about the derivation of Eqs. (5) and (6) can be found in Ref.^31^. In Eqs. (5) and (6), $\theta_{0}$ stands for fracture initiation angle and by solving the above equations for different fracture angles, the GMTS model fracture limit curve can be drawn. The value of $r_{c}$ can be derived for both the MTS and GMTS criteria using Eq. (7).

7 $$ r_{c} = \frac{1}{2\pi }\left( {\frac{{K_{Ic} }}{{\sigma_{u} }}} \right)^{2} $$

where $K_{Ic}$ and $\sigma_{u}$ are fracture toughness and ultimate tensile strength, respectively.

### Equivalent material concept (EMC)

**Figure 6:**
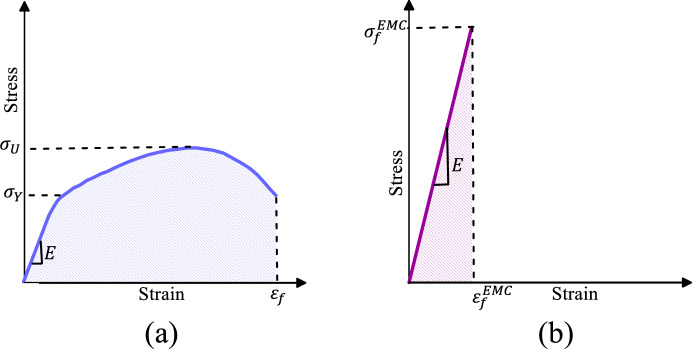
Stress–strain curves of (**a**) real elastoplastic and (**b**) virtual linear elastic material.

To avoid intricate and time-consuming elastoplastic analyses, Torabi^33^ suggested the EMC as an innovative solution. This approach entails the conversion of the actual ductile material into a fictitious brittle material. As a consequence, linear elastic criteria can be applied to assess the failure of an initially ductile substance. The foundation of this methodology rests on the premise that both the actual ductile material and the virtual linear elastic material share identical strain energy density (SED), as well as the same Young’s modulus as depicted in Fig. 6. With these assumptions in mind, the following equations can be used to determine the ultimate tensile strength of a virtual brittle material $\left( {\sigma_{f}^{EMC} } \right)$.

8 $$ \left( {SED} \right)_{EMC} = \left( {SED} \right)_{tot.} $$

9 $$ \sigma_{f}^{EMC} = \sqrt {2E\left( {SED} \right)_{EMC} } $$

where $\left( {SED} \right)_{tot.}$ is equal to the sum of the SED of the elastic and plastic regions of the actual ductile material. To establish a connection between EMC and the GMTS criterion, it is solely essential to incorporate the value of $\sigma_{f}^{EMC}$ within the equations associated with this criterion.

## Finite element modeling

An FE analysis was conducted to obtain experimental SIFs at the onset of fracture, as well as calculating geometry factors ($Y_{I}$ and $Y_{II}$) and dimensionless T-stress ($T^{*}$) which are needed to predict critical SIFs according to the criteria applied. The FE simulation was employed to determine the precise crack angle corresponding to the pure mode II loading condition, which is approximately 40.5°. Consequently, by utilizing the geometric factors ($Y_{I}$ and $Y_{II}$) and dimensionless T-stress values acquired through FE analysis for 10 distinct crack angles ranging from 0° to 40.5°, and solving Eqs. (5) and (6), the critical values of SIF for MTS and GMTS criteria were calculated.

**Figure 7:**
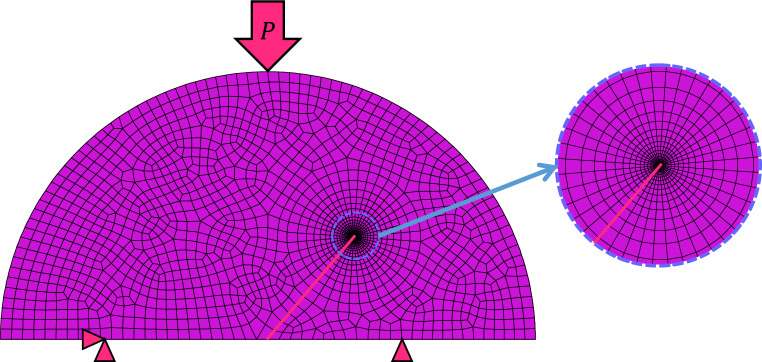
Sample mesh pattern of the SCB specimen.

The convergence of the analysis was enhanced by employing the 8-node quadratic plane stress quadrilateral elements. Figure 7 illustrates the FE model of an SCB sample with a crack angle of 20°. As depicted in the figure, the mesh size was refined near the crack tip to accurately capture the high stress gradient in this region.

## Results and discussion

This section presents the results of tensile and fracture tests, along with theoretical predictions of fracture limit curves and related discussions.

### Tensile test results

**Figure 8:**
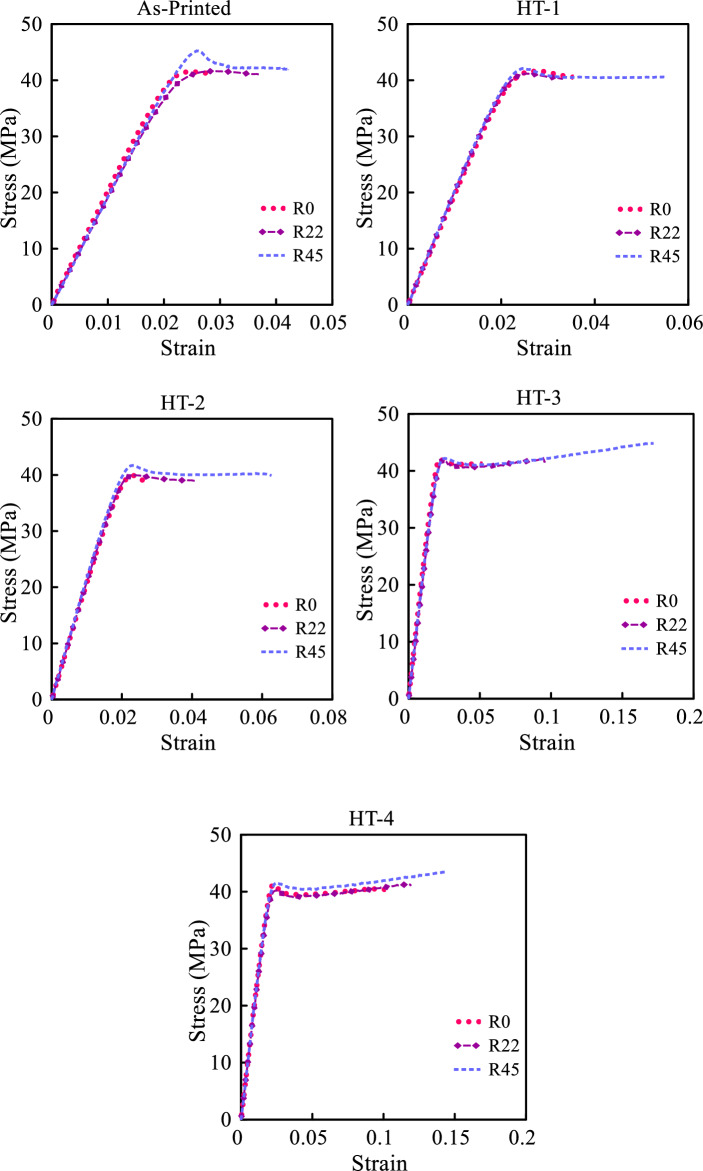
Stress–strain curves of as-printed and heat-treated (HT-1 to HT-4) samples.

Figure 8 illustrates the stress–strain curves of both as-printed and heat-treated samples for three distinct raster angles. R0, R22 and R45 represent the raster angles of 0/90°, 22/ − 68°, and 45/ − 45° respectively. It is clear from this figure that in all cases, both in the as-printed sample and in the annealed sample, the yield stress for the sample R45 with $\psi_{r} = 45/ - 45^\circ$ is slightly higher than that for the other two samples. Furthermore, samples R0 and R22 with raster angles of $\psi_{r} = 0/90^\circ$ and $\psi_{r} = 22/ - 68^\circ$ show a minor degree of plastic deformation accompanied by failure. Conversely, samples designed with a raster angle of $45/ - 45^\circ$ demonstrate remarkable ductility and a higher capacity to withstand deformation before failure. The differences in behavior highlight the importance of raster orientation in determining the mechanical responses of 3D-printed samples. To gain a deeper comprehension of this phenomenon, let’s take the specimen with the raster angle of $\psi_{r} = 0/90^\circ$ as an example.

In this particular case, half of the layers are fabricated with a zero-degree raster orientation relative to the applied load (i.e. parallel to the loading direction), while the remaining half are situated perpendicular to it. As a result, only half of the layers actively withstand the applied force, consequently influencing the overall strength of the specimen. This observation implies that areas located between rasters are comparatively less resistant to overall failure. Additionally, it is noticeable that the modulus of elasticity remains relatively uniform for all specimens with different raster angles, in contrast to the anisotropic (raster angle-dependent) nature exhibited by maximum elongation and toughness.

Upon closer inspection of the stress–strain curves depicted in Fig. 8, it becomes evident that the difference between the stress–strain curves associated with varying raster angles diminishes when heat treatment is employed. It appears that heat treatment can homogenize material properties regardless of the raster angle used in fabrication. Hence, heat treatment emerges as a viable method for enhancing the mechanical properties of additively manufactured components. Upon contrasting the stress–strain curves of both the as-printed and annealed samples, it becomes evident that the heat treatment procedure had a minimal impact on tensile strength, yet it notably amplified the energy absorption capacity.

### Fracture test results

**Figure 9:**
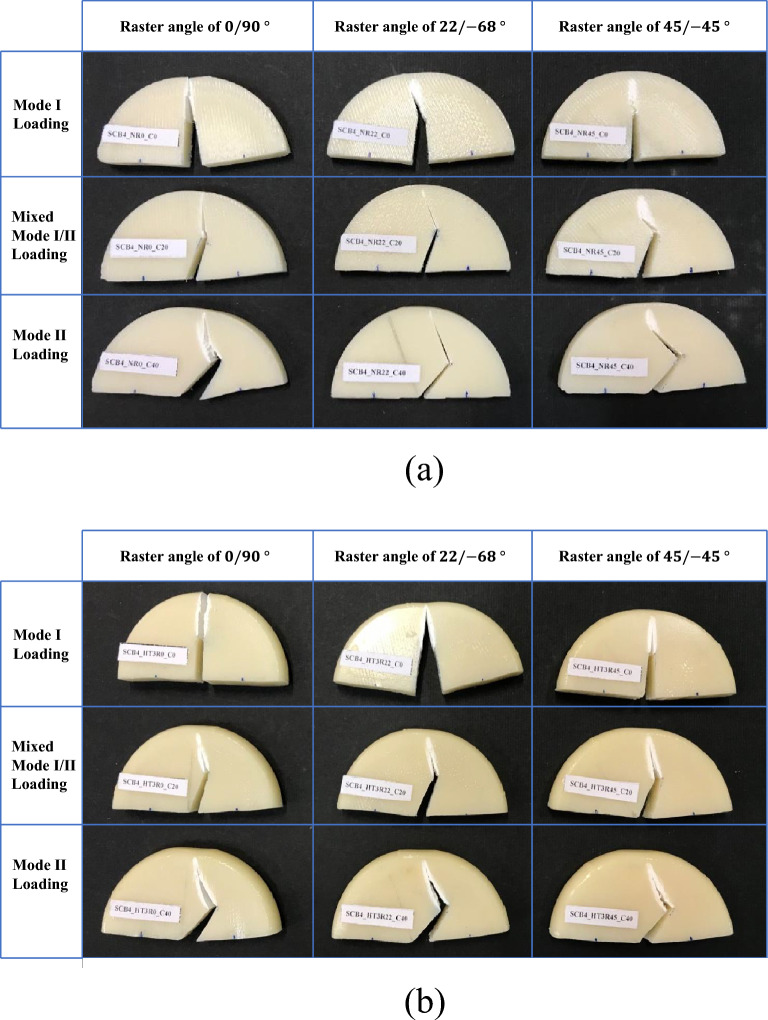
Broken (**a**) as-printed and (**b**) annealed SCB specimens.

To investigate the fracture initiation in the 3D-printed ABS parts, SCB samples made with different raster angles and heat treatments were subjected to conditions of pure mode I, mixed mode I/II, and pure mode II loading. Figure 9 shows the as-printed and heat-treated (HT-3) samples after fracture. The as-printed and heat-treated samples were labeled “N” and “HT3” in the picture, respectively. The labels also indicate raster angles of 0, 22, and 45 degrees respectively from the left column to the right. The first row of each picture shows the specimens that went through mode I loading, while the second and third rows indicate mixed mode and pure mode II conditions, respectively.

Based on the MTS criterion, an isotropic material subjected to pure mode I loading, mixed mode I/II loading, and pure mode II loading exhibits crack initiation angles of 0°, 42°, and 69°, whereas according to the GMTS criterion, these angles for the SCB samples are 0°, 44°, and 76°. However, it is noteworthy that the crack initiation angles in the as-printed samples do not align with the estimation of the MTS and GMTS theoretical models. Nevertheless, a notable shift occurs in the annealed samples, where the crack initiation angles align more closely with those established by the MTS and GMTS criteria. By comparing the crack growth paths obtained from the samples without heat treatment and those subjected to the post-processing, it becomes evident that the degree of anisotropy inherent in the 3D-printed samples diminishes with annealing. Consequently, the material behaves like isotropic materials, and crack growth is no longer affected by the raster pattern. In contrast, within as-printed samples, the crack growth path is substantially influenced by the raster angle. According to Fig. 9, samples with varying raster angles exhibit distinct behaviors under pure mode I loading. For instance, the sample with a 0/ − 90° raster angle exhibited crack growth as expected in an isotropic material, while the sample featuring a 22/ − 68° raster angle revealed crack propagation at an angle nearly equivalent to the raster orientation. Moreover, in the case of the 45/ − 45° raster angle sample, crack kinking was observed. The occurrence of crack kinking results in elevated energy absorption and heightened resistance to crack propagation. This phenomenon explains why an increased raster angle corresponds to greater resistance to failure within the sample. In pure mode II loading, an inverse phenomenon occurs, where the sample with raster angles of 45/ − 45° exhibits lower resistance to crack growth compared to the sample with a raster angle of 0/90°. This divergence arises because, in mode II, as per the predictions of MTS and GMTS theoretical models, the crack tends to propagate at an angle of approximately 69° or 72°. Consequently, in specimens with raster angles of 45/ − 45°, the crack can propagate between the rasters. In contrast, the sample with a raster angle of 0/90° necessitates crack propagation through the grids, leading to kinking, and thereby exhibiting greater resistance to failure.

**Figure 10:**
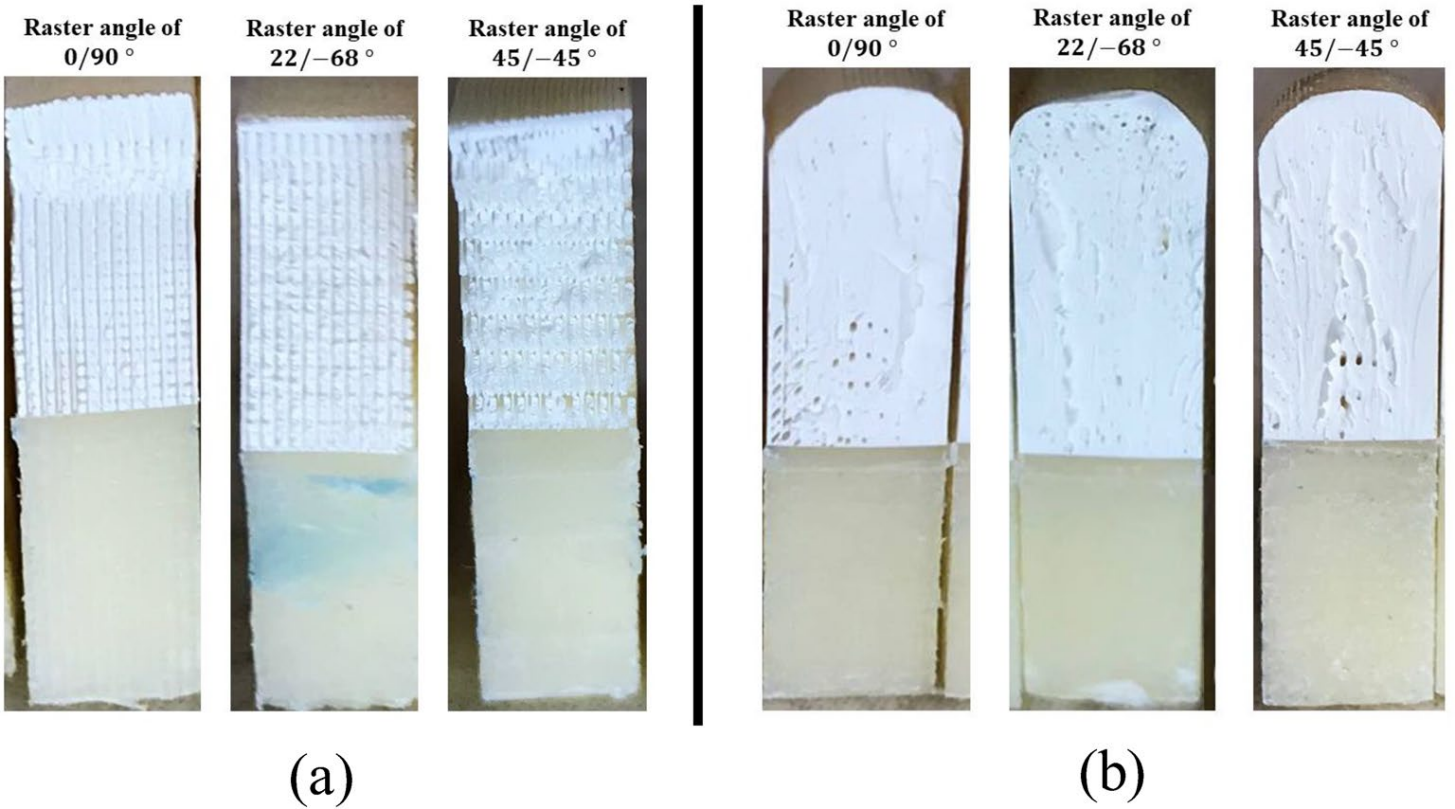
Mode I fracture surfaces of the (**a**) as-printed, and (**b**) annealed (HT-4) SCB samples.

**Figure 11:**
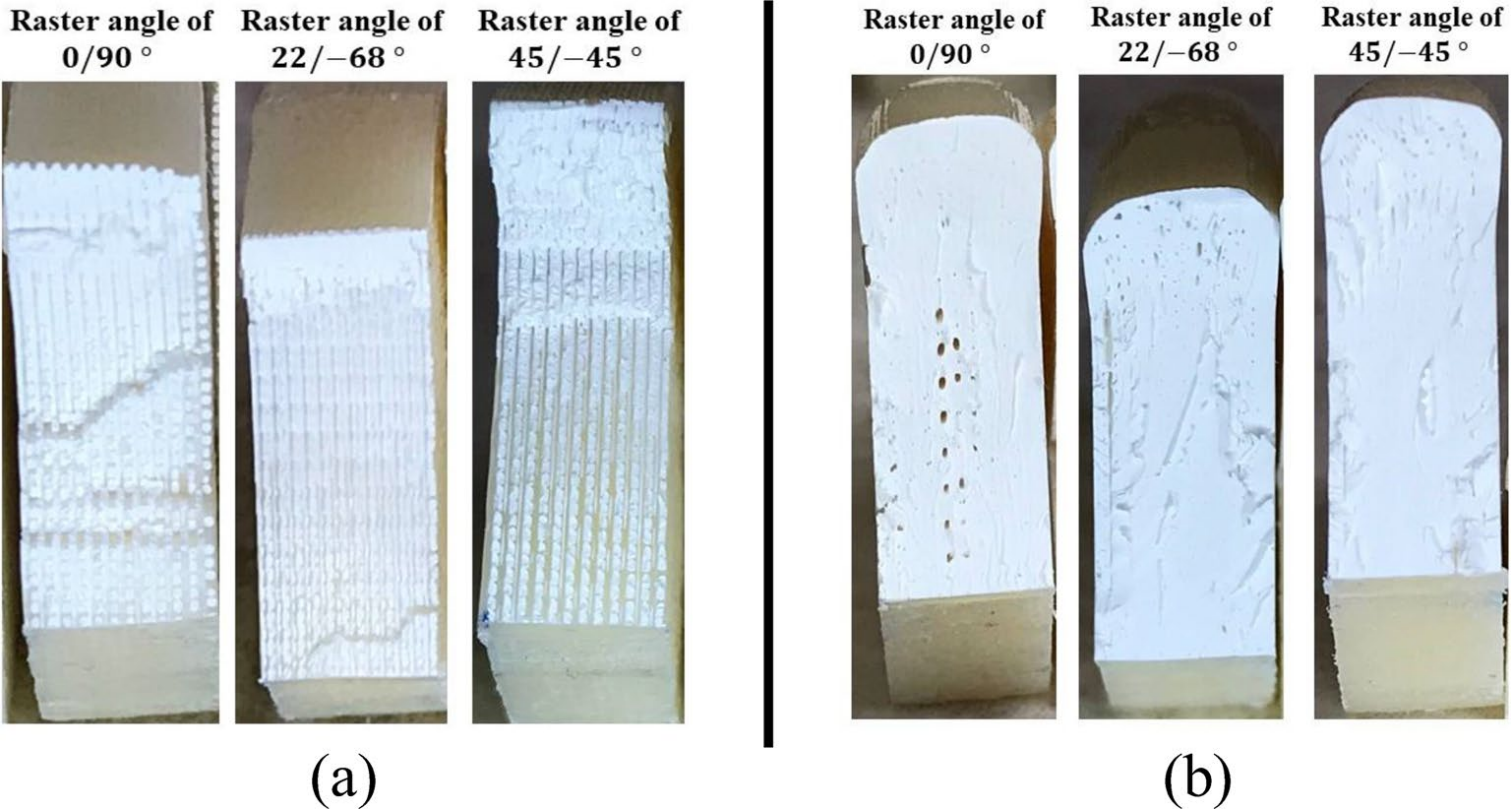
Mode II fracture surfaces of the (**a**) as-printed, and (**b**) annealed (HT-4) SCB samples.

Figures 10 and 11 depict close-up pictures of the fracture surfaces in normal and heat-treated cases under mode I and pure mode II loading, respectively. In the normal case of Fig. 10, the mode I smooth fracture surface of the 0/90° specimen indicates direct crack growth along the 90-degree raster layout, breaking inter-raster bonds. Similarly in the 22/ − 68° sample, the surface shows an inter-raster crack growth, while, for the 45/ − 45 case, the uneven fracture surface exhibits crack multi-kinking in which the crack interchangeably switches between breaking 45° and − 45° rasters. As a result, much more energy is absorbed in the latter case which explains its higher fracture toughness compared to the other two. Meanwhile, part (b) of Fig. 10 indicates no deviation from the expected mode I crack opening in homogeneous materials. The rough fracture surface in all heat-treated samples shows the higher crack resistance of these samples compared to the as-printed ones. In normal cases of Fig. 11, however, kinking happens in the 0/90° specimen while the 45/ − 45° shows an inter-raster fracture, explaining the higher mode II fracture resistance of the former. For heat-treated cases depicted in part (b) of Fig. 11, the rough surfaces for all rasters indicate a higher fracture toughness^16^. By taking a closer look at the fracture surfaces of these heat-treated samples, it becomes evident that while the annealing process rearranged the raster pattern, some central voids are still apparent in the material.

**Figure 12:**
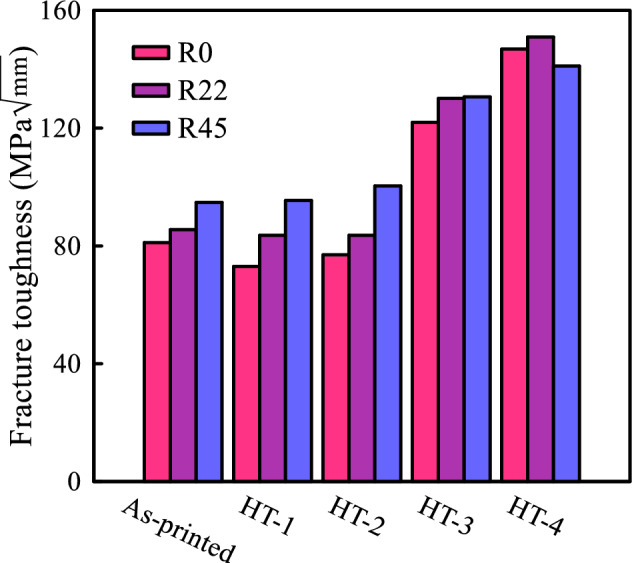
Fracture toughness values of as-printed and heat-treated samples with three different raster angles.

According to Fig. 12, heat treatment at 125 °C (i.e. HT-1 and HT-2) exerts a negligible impact on the fracture toughness, while heat treatment at 165 °C (i.e. HT-3 and HT-4) enhanced resistance to failure. It demonstrates the importance of precise calibration of the annealing process temperatures in order to attain optimal results. Furthermore, a comparison between two post-processing methods, HT-3 and HT-4, both conducted at identical temperatures, reveals that a rise in the annealing process pressure corresponds to increases in the fracture toughness.

### Theoretical prediction

Initially, an attempt was made to directly apply the linear elastic criteria of MTS and GMTS to assess the fracture of SCB samples fabricated from ABS material. However, this approach failed to yield satisfactory results. The rationale behind this result lies in the stress–strain curves illustrated in Fig. 8, which reveal that the ABS material, whether in its as-printed or annealed form, does not exhibit exclusively linear behavior but instead shows some degree of ductile behavior. Meanwhile, employing non-linear criterion proves to be complicated and time-consuming. Hence, integrating EMC with linear elastic fracture criterion provides an avenue for improving results without going through the complexities and time constraints associated with non-linear methods. Table 3 gives the $\sigma_{f}^{EMC}$ values calculated using stress–strain curves and Eq. (9). The significantly elevated ultimate stress strength of the virtual material following HT-3 heat treatment suggests an increase in toughness, characterized by a larger area under the stress–strain curve, and enhanced energy absorption capacity of the sample.

**Table 3:** Average ultimate tensile strength of virtual brittle material.

Specimen raster angle condition	$\sigma_{f}^{EMC}$(MPa)				
	Normal	HT-1	HT-2	HT-3	HT-4
0/90°	58.51	60.79	53.53	112.59	95.34
22/ − 68°	58.32	59.96	63.29	120.89	102.5
45/ − 45°	64.23	87.15	100.64	130.54	138.3

**Figure 13:**
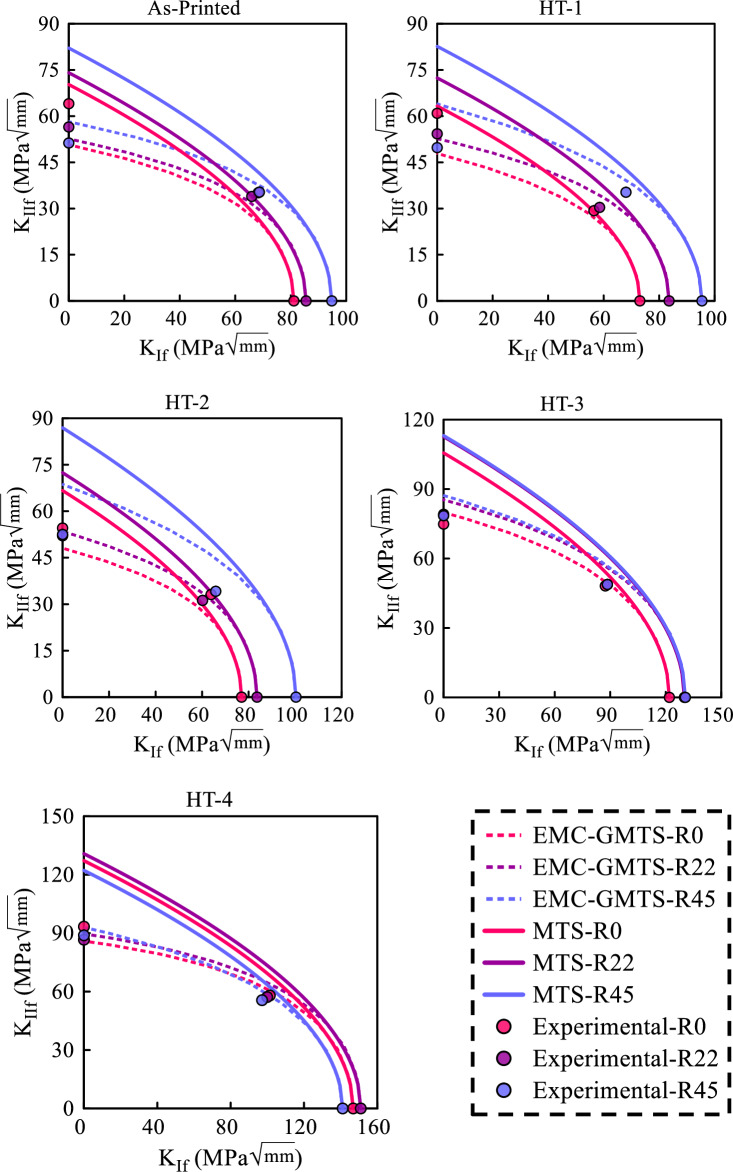
Fracture limit curves of MTS and EMC-GMTS criteria along with average experimental measurements.

Utilizing the $\sigma_{f}^{EMC}$ values outlined in Table 3 and substituting them into Eq. (7) to compute $r_{c}$, and subsequently, by simultaneously solving Eqs. (5) and (6), the fracture limit curves can be derived for the GMTS criterion. Figure 13 shows the fracture limit curve of the MTS linear elastic criterion and the EMC-GMTS combined criterion as well as the average experimental observations for samples made at three distinct raster angles.

Figure 13 illustrates how heat treatment minimizes the variation in fracture toughness across samples with different raster angles. This becomes particularly pronounced as the annealing process temperature and pressure levels are elevated. Increased temperature and pressure during annealing contribute to homogenizing fracture toughness characteristics of the samples. Furthermore, it becomes evident that as the temperature and thermal operating pressure are raised, there is a corresponding increase in the critical SIFs values for both mode I and mode II loading conditions. This phenomenon is attributed to the strengthening of the bond between the rasters during the heat treatment process.

Upon comparing the fracture limit curves derived from the MTS and EMC-GMTS theoretical models, it becomes evident that the combined EMC-GMTS approach offers superior accuracy across all scenarios. The superiority of the EMC-GMTS model can be attributed not solely to its incorporation of the T-stress term but also to its utilization of the EMC, which transforms the actual ductile material into a virtual brittle material.

The experimental observations of as-printed parts depicted in Fig. 13 lead to the conclusion that samples constructed with a raster orientation of $\psi_{r} = 45/ - 45^\circ$ exhibit the highest critical SIF when subjected to pure mode I loading, whereas these samples display the lowest critical SIF under pure mode II loading conditions. However, an inverse pattern is observed in samples with a 0/90° raster angle. This observation underscores the necessity of employing a fracture limit band rather than a singular fracture limit curve due to the inherent anisotropy of 3D-printed samples. In this context, the lower limit of this band corresponds to the fracture limit curve associated with the weakest raster angle (in this case, the 0/90° raster angle), while the upper limit corresponds to the strongest raster angle (here, the 45/ − 45° raster angle). When considering this approach, it becomes evident that nearly all the experimental results fall within the bounds of these two fracture limit curves. Furthermore, it is apparent that by implementing heat treatment and reducing the degree of anisotropy in the samples, particularly in the HT-3 and HT-4 processes, the separation between the curves representing the strongest and weakest raster angles diminishes, causing them to overlap. A similar phenomenon concerning the effect of layer orientation on the fracture of 3D-printed specimens under combined mode I/II loading has been reported by Bahrami et al. in Ref.^34^.

## Concluding remarks

This study assessed the influences of the raster angle, as well as the annealing post-processing, on the mechanical characteristics and fracture behavior of ABS specimens made by an FDM 3D printer. Simple tensile test and in-plane mixed-mode fracture test were performed on samples made with three distinct raster angle configurations and subjected to four different heat treatments. The failure of additively manufactured parts was predicted by both the MTS linear elastic criterion and the combined EMC-GMTS criterion and their results were compared with experimental observations. Here are the findings of this study:

- Although changing the raster angle has a limited impact on Young’s modulus of ABS samples, it is noteworthy that adjusting the raster angle from 0/90° to 45/ − 45° leads to an improvement in the plastic deformation capacity of the sample, ultimately influencing the failure point.
- Elevating the raster angle within the as-printed sample results in enhancement of the critical SIF in pure mode I, while concurrently leading to a reduction in critical SIF in pure mode II. On the other hand, raising the temperature and pressure conditions of the annealing procedure increased the critical SIF of modes I and II loading conditions.
- In the as-printed sample, it is evident that the crack initiation angle is influenced by the raster orientation. However, in the heat-treated samples, the impact of the raster angle diminishes, and the material’s behavior aligns more closely with that of an isotropic material.
- The combined EMC-GMTS criterion, owing to its incorporation of the T-stress term and the application of the EMC to transform the original ductile material into a virtual linear elastic material, demonstrated an acceptable ability to properly estimate the fracture of 3D-printed ABS samples exposed to mixed-mode in-plane loading conditions.

## Future work

For the current study, it was assumed that the fracture toughness remained constant throughout the specimen’s thickness (plane stress condition). Additionally in the theoretical approach, the material was considered homogeneous. The specimen’s fracture behavior was modeled using linear elastic fracture mechanics along with the equivalent material concept to obtain the most precise results while avoiding overly complicated nonlinear calculations. The assumptions in the production of the samples include the negligible effect of room temperature and humidity change regarding the fabrication of different samples at different times of the day, and the consistency of quality in the filament material used. Furthermore, in the heat treatment process, it was assumed that the pressure on top of the specimens was maintained constant and evenly distributed throughout the process, and again the influence of room temperature on the temperature inside the furnace was insignificant.

Some valuable related areas to conduct further research on include:

1. Utilization of different heat treatment methods on the AM materials, such as investigating the effect of annealing duration, devising another post-processing method, such as hot isostatic pressing (HIP), or any post-processing that intends to affect a specific property, e.g. environmental resistance.
2. Researching the dimensional accuracy of heat-treated AM parts and suggesting means to overcome its limitations. Since dimensional alteration was observed in the current study, this would be beneficial to be considered either through design optimization or minimizing dimension alteration due to heat treatment.
3. Investigating the effect of post-processing on materials produced by other AM methods, such as multi-material FDM, carbon fiber reinforced FDM, etc., and studying how different material combinations react to post-processing treatments.
4. Implementing cutting-edge computational methodologies and advanced machine learning algorithms will enable the accurate prediction of failure characteristics in 3D printed components across various loading scenarios and post-processing treatments. Moreover, this innovative approach will unravel the intricate interplay between construction parameters and mechanical properties, leading to a deeper understanding and optimization of additive manufacturing processes.

This study demonstrates that the fracture toughness and energy absorption capacity of 3D-printed parts can be significantly enhanced through heat treatment at optimal temperatures and pressures. However, this enhancement comes with slight changes in initial dimensions, which can be addressed through trial-and-error design optimizations. Consequently, this post-processing method can be readily applied in practical applications to preserve the favorable mechanical properties of ABS material while benefiting from the rapid prototyping capabilities of AM.

## Data Availability

The datasets used and/or analyzed during the current study are available from the corresponding author upon reasonable request. (B.B.: bahramibahador@gmail.com).
